# Redox-based epigenetic status in drug addiction: a potential contributor to gene priming and a mechanistic rationale for metabolic intervention

**DOI:** 10.3389/fnins.2014.00444

**Published:** 2015-01-22

**Authors:** Malav S. Trivedi, Richard Deth

**Affiliations:** Department of Pharmaceutical Sciences, Northeastern UniversityBoston, MA, USA

**Keywords:** glutathione, s-adenosylmethionine, EAAT3, drug addiction, withdrawal, gene priming, N-acetylcysteine

## Abstract

Alcohol and other drugs of abuse, including psychostimulants and opioids, can induce epigenetic changes: a contributing factor for drug addiction, tolerance, and associated withdrawal symptoms. DNA methylation is a major epigenetic mechanism and it is one of more than 200 methylation reactions supported by methyl donor S-adenosylmethionine (SAM). Levels of SAM are controlled by cellular redox status via the folate and vitamin B12-dependent enzyme methionine synthase (MS). For example, under oxidative conditions MS is inhibited, diverting its substrate homocysteine (HCY) to the trans sulfuration pathway. Alcohol, dopamine, and morphine, can alter intracellular levels of glutathione (GSH)-based cellular redox status, subsequently affecting SAM levels and DNA methylation status. Here, existing evidence is presented in a coherent manner to propose a novel hypothesis implicating the involvement of redox-based epigenetic changes in drug addiction. Further, we discuss how a “gene priming” phenomenon can contribute to the maintenance of redox and methylation status homeostasis under various stimuli including drugs of abuse. Additionally, a new mechanistic rationale for the use of metabolic interventions/redox-replenishers as symptomatic treatment of alcohol and other drug addiction and associated withdrawal symptoms is also provided. Hence, the current review article strengthens the hypothesis that neuronal metabolism has a critical bidirectional coupling with epigenetic changes in drug addiction exemplified by the link between redox-based metabolic changes and resultant epigenetic consequences under the effect of drugs of abuse.

## Introduction

Drug and alcohol dependence are debilitating neuropsychiatric disorders with multi-factorial and complex etiology, associated with high morbidity and mortality rates. Research over the past decade has indicated a strong involvement of several molecular pathways of learning and memory in drug addiction (Nestler, 2002). Stable changes in gene expression under the influence of drugs of abuse are believed to mediate, at least in part, the transition from a recreational drug user to a drug addict, by inducing neuronal adaptations in morphology and synaptic plasticity (e.g. long-term potentiation, long-term depression) (Nestler, 2002). Mounting evidence also indicates that epigenetic changes, specifically alterations in the pattern of DNA and histone methylation, can produce long lasting alterations in gene expression, which affects behavior (Tsankova et al., 2007; Renthal and Nestler, 2008; Maze and Nestler, 2011). Indeed, several studies suggest that epigenetic-mediated changes in gene expression might be an important contributor to drug tolerance and dependence (Chao and Nestler, 2004; Hyman et al., 2006; Maze and Nestler, 2011; Robison and Nestler, 2011). Further, the stochastic nature of these periods of gene sensitization/imprinting might be revealed and even amplified during drug abstinence, contributing to behavioral withdrawal symptoms.

The best-understood and most stable epigenetic modification is methylation of cytosine nucleotides in DNA, which regulates the transcriptional plasticity of mammalian genomes (Bellizzi et al., 2013). DNA methylation is carried out by DNA methyltransferase (DNMT) enzymes and occurs primarily where a cytosine (C) precedes a guanine (G) in the DNA sequence [“C—phosphate link—G—,” or cytosine–guanine dinucleotides (CpG)], although cytosine methylation at non-CpG positions has also been reported (Lister et al., 2009). Methylation of DNA is facilitated and regulated by levels of the methyl donor S-adenosylmethionine (SAM) and the metabolic pathways which affect the level of SAM.

**Figure 1:**
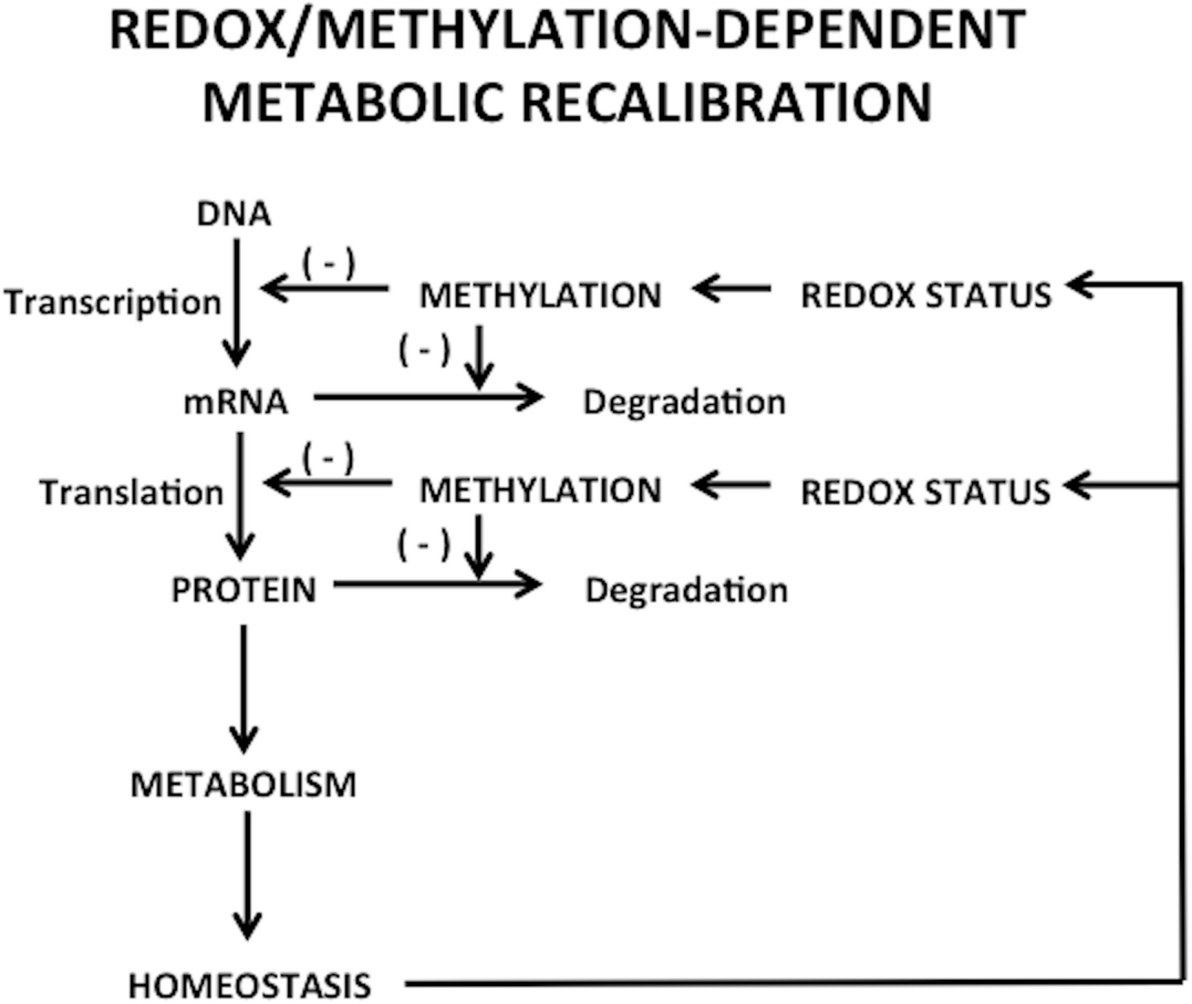
**Bidirectional regulation between transcriptional/epigenetic changes and metabolic homeostasis**.

An important feature of metabolic processes is the transcriptional regulation of rate-limiting metabolic enzymes, usually mediated by epigenetic mechanisms. Moreover, the activities of various enzymes involved in epigenetic modifications, including DNMTs and histone methyltransferase, are regulated, in part, by the concentrations of required substrates and cofactors via feed-back networks (Lee and Workman, 2007). Hence, on one side, epigenetic changes can affect metabolism by regulating the expression of metabolic enzymes (Wolf et al., 2011), while on the other side, metabolism can disturb epigenetic status, resulting in changes in gene expression or chromatin structure (Wellen and Thompson, 2012), which might serve as a compensatory mechanism altering transcription in response to changes in the cellular environment. Thus, the cell's metabolic state has complex and bidirectional integration with the epigenome and transcriptional regulation, as depicted in Figure 1.

**Figure 2:**
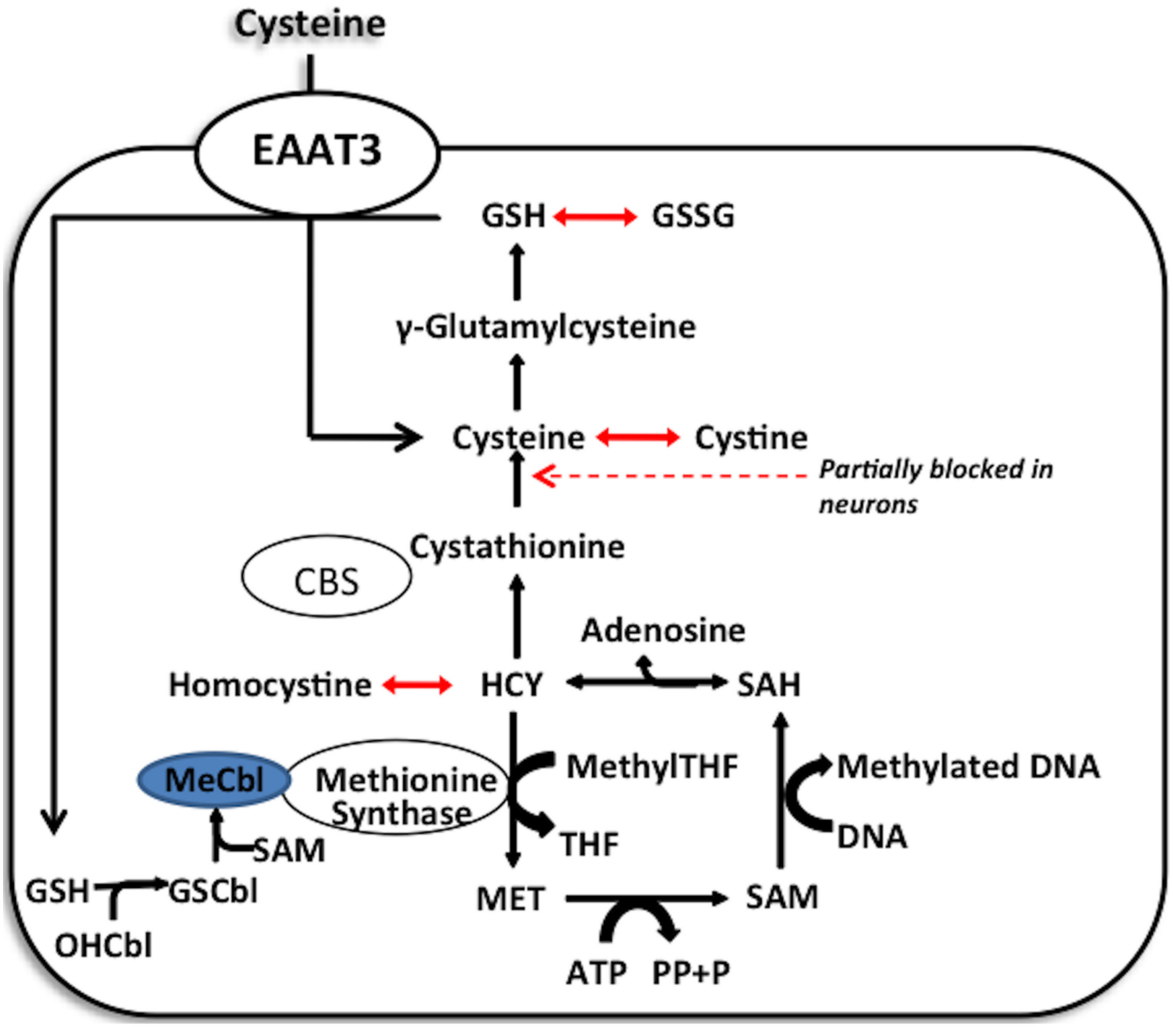
**Methionine synthase acts as a redox switch**. Methionine synthase contains a redox-active methylcobalamin cofactor. Under oxidative stress, this cofactor becomes oxidized, limiting methionine synthase activity. Under these conditions, homocysteine can be condensed with serine to form cystathionine and cysteine, which supports GSH synthesis. Only when cellular redox state is restored does the favorable GSH/GSSG ratio allow for the glutathionylation of oxidized cobalamin and methylation of the glutathionylcobalamin to reactivate the enzyme.

Cellular redox status exerts control over methylation through the folate and vitamin B12-dependent enzyme methionine synthase (MS), whose activity controls the ratio of the methyl donor SAM to the methylation inhibitor S-adenosylhomocysteine (SAH). Under oxidative conditions MS is inhibited, diverting its substrate homocysteine (HCY) to the transulfuration pathway. Availability of reduced glutathione (GSH) to donate electrons to oxidized proteins or lipids determines the intracellular redox status, reflected as the ratio of GSH to its oxidized dimeric form (GSSG). Cysteine, the rate-limiting precursor for the synthesis of GSH, is made available by two pathways: (1) extracellular uptake or (2) metabolism of homocysteine via the transulfuration pathway. However, the transulfuration pathway is limited in adult cortical neurons and about 90% of all available intracellular cysteine, is transported by the excitatory amino acid transporter 3 (EAAT3). Thus, EAAT3 plays an extremely important role in maintaining redox status and subsequently the activity of more than 200 methylation reactions, including DNA and histone methylation (Figure 2).

Opioids, exemplified by morphine, can induce global DNA hypomethylation and affect retrotransposon transcription levels by transiently inhibiting cysteine uptake and decreasing intracellular GSH levels (Trivedi et al., 2014a). Here, the current evidences are compiled to present, how the effects of drugs of abuse, including alcohol, might commonly impinge on EAAT3-mediated cysteine uptake by different mechanisms in neuronal cells, consequently affecting redox status, MS activity, and methylation reactions, including methylation of DNA.

## Drugs of abuse and glutathione-based redox homeostasis

Although pharmacological actions and downstream signaling pathways consequent to administration of alcohol and other drugs of abuse have been described, the nature of biochemical and metabolic changes contributing to the development of chronic effects, drug dependence, and withdrawal syndrome are less known. Alcohol and other drugs of abuse are reported to alter redox status, and we will focus on their effects on GSH-based redox status, ROS species, and how these changes affect the metabolite status for various other elements and key intermediates of the transulfuration pathway and one-carbon metabolism, finally affecting SAM levels as well as epigenetic status.

The relationship between drugs of abuse and redox/methylation status is not coincidental. These drugs act on pathways, which stimulate attention, awareness, and memory formation, and these mechanisms stimulate neurons to grow axons and dendrites and form new synapses, as well as induce axon pruning and synapse retraction (Nestler, 2002; Chao and Nestler, 2004). However, these synapses, dendrites and axons are maintained at the cost of an underlying metabolic demand (Xu et al., 2003). Neurons require ATP to maintain the ion gradient needed for each action potential sent along the axon, and that ATP production induces ROS generation and accumulation, representing a demand on antioxidant resources (Deavall et al., 2012). This can lead to downstream compensatory adaptive responses in the transulfuration pathway as well as the methylation capacity of the cells, which can induce global epigenetic changes, increasing transcription of several redox-pathway related genes including EAAT3 (Trivedi et al., 2014a). This tightly linked control of antioxidant and neuronal activity might be a way of ensuring a narrow window of redox potential in the brain. EAAT3 transcription is linked to redox status through the Keap1-Nrf2 pathway (Dong et al., 2008; Escartin et al., 2011). When redox-sensitive cysteine residues on Keap1 become oxidized, Nrf2 disassociates and causes an increase in the transcription of EAAT3, but not the other EAATs, although activation of Nrf2 is not the only mechanism increasing the surface expression of EAAT3 (Escartin et al., 2011). The important role of EAAT3 in regulating redox status in the human brain is well-recognized, and its targeting by drugs of abuse can link their addictive potential to both ROS production and mitigation. However, other glutamate transporters namely GLT1 (glutamate transporter 1) and xCT^−^ (catalytic subunit of the cystine–glutamate exchanger), are also reported to be involved in development of drug addiction. In fact, a decrease in expression of GLT-1 and xCT in nucleus accumbens (NA) is the most reliable findings across drugs of abuse and administration protocols found following self-administration of cocaine, heroin, alcohol, and nicotine (Knackstedt et al., 2009; Sari and Sreemantula, 2012; Gipson et al., 2013; Reissner et al., 2014). Activation of GLT1 by MS-153, a cerebroprotective agent, can effectively inhibit the induction of conditioned place preference to methamphetamine (MA), cocaine, and morphine (Nakagawa et al., 2005, 2001). Although, neurocircuitry of the glutamatergic system contributes to alcohol and drug addiction and EAAT3 is classified as a glutamate transporter, EAAT3 provides about 60–80% of cysteine uptake in neurons and its influence over redox and epigenetic status is the focus of this review.

### Opioids

Neuronal cell membranes are composed of phospholipids, glycolipids, cholesterol, and proteins, and drugs of abuse, including opioids, are reported to induce lipid peroxidation (Adibhatla and Hatcher, 2010) (compromising neuronal membrane integrity) and oxidative DNA damage, leading to neuronal apoptosis, neurotoxicity, and neurodegeneration. Opioids can induce untoward effects due to biochemical alterations in target cells (Christie, 2008). Heroin and morphine deplete the antioxidant GSH in peripheral tissues, and recent reports also document pro-oxidant effects of morphine in the central nervous system (CNS) (Martin and Fraser, 1961; Bhat et al., 2004; Guzmán et al., 2006; Xu et al., 2006; Mannelli et al., 2009; Gutowicz et al., 2011), along with alterations in antioxidant defense in animals and humans after administration or withdrawal from these agents (Xu et al., 2003; Tegeder and Geisslinger, 2004; Mannelli et al., 2009). Intracerebroventricular administration of morphine is associated with significantly decreased GSH levels in cerebrospinal fluid samples, suggesting that the central nervous system would be vulnerable to damage from oxidative stress (Goudas et al., 1999). Morphine and heroin tolerance has also been shown to occur in conjunction with a concomitant decrease in the levels of EAAT3. Continuous morphine exposure leads to an increase in the ubiquitinylation and subsequent degradation of surface-expressed EAAT3 (Mao et al., 2002; Yang et al., 2008a,b). Since EAAT3 transports cysteine necessary for GSH synthesis, ubiquitinylation, and degradation of EAAT3 might result in decreased levels of cysteine in the presence of opioids like morphine, as indicated by our recent study. These changes in cysteine uptake are also translated into changes in the redox and methylation capacity of the cell, along with subsequent changes in global DNA methylation (Trivedi et al., 2014b), as well as changes in retrotransposon methylation (i.e., long interspersed nuclear element 1 (LINE1) methylation Trivedi et al., 2014a). Further, redox status can also regulate mRNA methylation levels as well as mRNA translocation from nucleus to axonal cones, thereby regulating neuronal morphology and local synaptic mRNA transcript levels (Trivedi and Deth, 2012).

### Alcohol

Both clinical and animal model studies have demonstrated diverse effects of chronic ethanol exposure on regulatory enzymes and metabolites involved in methionine metabolism, indicating elevated homocysteine and SAH levels, accompanied by a reduction in SAM and GSH levels (Kim et al., 2003; Augustyniak et al., 2005; Waly et al., 2011). As illustrated in Figure 1, reduced activity of either MS or CBS can induce elevated levels of homocysteine, whereas reduced activity of MS or MAT can decrease SAM levels. Since SAHH is reversible, elevated levels have SAH and a decline in the SAM:SAH ratio could also result from decreased conversion of SAH to HCY or increased conversion of homocysteine into SAH. Decreased mRNA levels of MS, MAT, and CBS are reported in studies of liver biopsies from ALD patients, and concurrent studies also report lower activities of MS, MAT, and SAHH, along with increased glycine-N-methyltransferase in chronic ethanol-fed pigs, which correlates to the protein levels of these enzymes evaluated from the histopathological evaluation from ALD patients (Villanueva and Halsted, 2004). Decreased MS activity, with a compensatory rise in BHMT activity has been reported in ethanol-fed rats (Barak et al., 1987). Both MAT1A, expressed in adult liver, and MAT2A, expressed in fetal liver and extra-hepatic tissues, can convert methionine to SAM. However, MAT1A is susceptible to inactivation by ROS generated by ethanol-induced elevated CYP2E1, due to nitrosylation or oxidation of amino acid residues (Sánchez-Góngora et al., 1997; Ruiz et al., 1998). Interestingly, since SAM promotes production of the antioxidant GSH by activation of CBS (Finkelstein et al., 1975; Prudova et al., 2006), the ethanol-induced and ROS-mediated inactivation of MAT1A expression can lead to decreased GSH production. A previous study from our lab showed that the ethanol-induced decrease in OHCbl-based MS activity is secondary to decreased GSH levels resulting in a decreased ability to synthesize MeCbl (Waly et al., 2011). Additionally, as discussed later, the ability of MeCbl to completely offset ethanol-mediated methionine synthase inhibition in brain cortex, but not liver, suggested tissue-specific differences in the GSH-dependent regulation of MS activity (Waly et al., 2011).

### Psychostimulants

Amphetamine and MA are highly addictive psychostimulants, causing potent central nervous system stimulant effects, generally accompanied by decreased appetite, hypothermia, paranoia, aggression, and a heightened sense of pleasure (Homer et al., 2008). Chronic use of MA causes neuronal loss (Ernst et al., 2000), associated with reductions in dopaminergic and serotonergic function, generally characterized by depletion in the levels of dopamine transporter (DAT), serotonin transporter (SERT), serotonin (5-HT), and dopamine (DA) levels (Krasnova and Cadet, 2009); however, the exact mechanism of action still remains unclear. Increasing evidence suggests that MA-induced neurotoxicity involves reactive oxygen species (ROS) and reactive nitrogen species (RNS) (Stephans and Yamamoto, 1994) and activation of downstream oxidative stress mechanisms. Moreover, amphetamine and MA formation of superoxides and hyponitrites, lead to neurodegeneration (Carvalho et al., 1999; 2001). These studies also report a significant decline in GSH levels under the influence of MA (Carvalho et al., 1999; 2001). Dopaminergic neurons are markedly decreased by early depletion of GSH, possibly mediated via induction of autophagy, accompanied by up-regulation of oxidative stress markers, namely 3-NT and 4-HNE, and N-acetylcysteine (NAC), a precursor for GSH synthesis, rescues MA-induced neurotoxicity and neurodegeneration (Chandramani Shivalingappa et al., 2012). Hence, alcohol and other drugs of abuse can induce ROS production and deplete levels of GSH antioxidant, as well as induce changes in SAM levels and methylation capacity, consequently inducing neuronal adaptations to maintain redox and methylation homeostasis, resulting in chromatin modifications and transcriptional changes as discussed next.

## SAM-dependent regulation of epigenetic status by drugs of abuse

Chromatin remodeling is important for mediating transcriptional responses and, in the case of neuronal function, it is suggested to contribute to various psychiatric disorders, including depression and schizophrenia (Guidotti et al., 2011; Peter and Akbarian, 2011; Robison and Nestler, 2011). In addition, studies have also shown that epigenetic changes contribute to behavioral abnormalities associated with drug addiction (Hyman et al., 2006; Maze and Nestler, 2011; Robison and Nestler, 2011). For example, repeated exposure to drugs of abuse induces long-lasting gene expression changes in the key brain reward region, the NA (Maze et al., 2011). There are many review and research articles which discuss this topic in-depth (refer to papers by Dr. Eric Nestler and his group Maze and Nestler, 2011; Robison and Nestler, 2011); however, some of the key points related to redox-related metabolic events will be discussed here.

### Alcohol

As mentioned previously, DNA/histone methylation can be directly regulated by SAM levels and indirectly by the pathways regulating SAM levels. The liver is the main source of SAM (also known as SAMe and AdoMet) biosynthesis and consumption, turning over nearly 8 g/day in a normal adult (Mudd et al., 1980). When SAM is depleted, homocysteine flux shifts toward remethylation to regenerate SAM, whereas when SAM levels are high, homocysteine is channeled toward the transulfuration pathway. Patients with liver disease, including alcohol-mediated liver damage, have multiple abnormalities in this pathway, resulting in lower SAM and GSH levels and a higher homocysteine level (Lu et al., 2002). Additionally, decreased hepatic SAM biosynthesis in ALD can also impact on methylation and antioxidant defense (Lu et al., 2002), reflected as decreased DNA methylation and lower GSH levels. This correlation has been demonstrated in rats fed intragastric ethanol for 9 weeks, which resulted in decreased hepatic levels of methionine, SAM, GSH, and DNA methylation by ~40% (Lu et al., 2000). As noted above, DNA hypomethylation affects chromatin structure, resulting in altered gene expression, rendering affected regions more accessible to DNA-damaging agents (Pogribny et al., 1995). One such example is hypomethylation of the *c-myc* gene and subsequent increase in *c-myc* expression, which results in increased genome-wide DNA strand breaks (Pogribny et al., 1995). Although, the cellular and molecular mechanisms associated with alcohol tolerance, dependence, and sensitivity are still not clearly identified, one of the important pharmacological targets of ethanol in the CNS is the NMDA receptor (reviewed by Kumari and Ticku, 2000). Chronic exposure to ethanol elevates brain NMDAR binding receptor density (Grant et al., 1990; Gulya et al., 1991), as well as mRNA levels and protein expression of NR2B subunit (Follesa and Ticku, 1995; Kalluri et al., 1998; Chandler et al., 1999; Bao et al., 2001). Altered NMDAR-mediated responses are proposed to contribute to the hyperexcitability and excitotoxicity associated with ethanol-withdrawal seizures (Thomas and Morrisett, 2000). Importantly, recent work in mouse cultured cortical neurons implicates epigenetic modifications as an important regulatory mechanism for transcription of NR2B gene. Intronic CpG methylation changes modulating NR2B gene expression are also reported under the influence chronic ethanol exposure (Marutha Ravindran and Ticku, 2004, 2005). Hence, although the work is in the early stages, it is already evident that alcohol can alter SAM levels, resulting in altered transcriptional status (e.g. NMDA receptor) and subsequent behavioral effects (e.g. tolerance and withdrawal) mediated via epigenetic changes.

### Psychostimulants and opioids

Similar to the effects of alcohol, psychostimulants like cocaine and MA, as well as opiates like morphine and heroin, can affect the enzymes catalyzing the addition or removal of post-translational modifications on histone tails (Sanchis-Segura et al., 2009; Maze et al., 2010, 2011; Jing et al., 2011; Sheng et al., 2011; Rehni et al., 2012). There are various modifications on histone tails including methylation, phosphorylation, and acetylation, but for the purpose of this paper we would focus on methylation of histone, as it is similar to DNA methylation in being directly regulated by the levels of SAM and the SAM:SAH ratio. Histone and DNA methylation levels can regulate normal cognitive function, and dysregulation has been implicated in several psychiatric disorders including drug addiction (Tsankova et al., 2007; Peter and Akbarian, 2011). Dimethylation of histone H3 at lysine 9 (H3K9me2) across the entire genome is catalyzed by enzyme G9a, a core subunit of a multimeric repressive histone lysine methyltransferase (KMT) complex (Fritsch et al., 2010; Shinkai and Tachibana, 2011). This complex, including G9a, plays a crucial role in regulating H3K9me2 in cocaine-induced transcriptional and behavioral plasticity changes, as well as the consequent regulation of susceptibility to chronic stress by prior cocaine exposure (Maze et al., 2010). Similarly, chronic morphine down-regulates H3K9me2 in NA across several different classes of repetitive elements, including LINE1 (Sun et al., 2012). However, the functional implications of this repressive histone methylation under the influence of opiates are not yet characterized. As indicated earlier, we also showed that morphine alters the DNA methylation levels in LINE1 retrotransposons (Trivedi et al., 2014a). Regulation of G9a/H3K9me2 in NA by chronic morphine as well as cocaine treatment indicates an integral role for G9a as part of dynamic repressive machinery in neurons for maintaining normal patterns of transcription and preventing aberrant gene expression under the influence of drugs of abuse or in response to negative environmental stimuli. The pathological down-regulation of this repressive machinery after chronic exposure to morphine or other emotional/environmental stimuli including other drugs of abuse, can lead to aberrant transcriptional control that contributes to abnormal synaptic plasticity, behavioral adaptations, and eventually symptoms observed in drug addiction (e.g. tolerance and withdrawal Robison and Nestler, 2011).

## Non-CpG methylation and heterochromatin

Changes mediated by drugs of abuse on redox status resulting in CpG methylation and epigenetic consequences are discussed. However, previous reports the role of GSH- and SAM-dependent metabolic regulation on non-CpG sites or non-cytosine methylation, including regulatory role of redox status on the pre-transcriptional and post-translational modifications of mRNA including splicing (Trivedi and Deth, 2012). Although mRNA molecules do contain methylcytosine, the major methylation of mRNA occurs with the methyl group at the sixth position on adenosine (m6A) (Pan, 2013). These methyl marks on mRNA can regulate the transcriptional state of mRNA. Additionally, methylation marks on RNA-binding proteins, e.g. Fragile-X syndrome related protein (FMRP), are also regulated by the levels of SAM (Dolzhanskaya et al., 2006). FMRP is involved in the dendritic and neurite transfer of mRNA molecules and a deficiency of FMRP function can lead to altered neurite levels and synaptic cleft levels of mRNA molecules under the effect of a stimuli, for example in neurodevelopmental disorders (Darnell et al., 2011) or under the effects of drugs of abuse (Smith et al., 2014). Similar to mRNA, the methylation levels of proteins are also regulated by SAM. For example, protein arginine-methyl transferase (PRMT), which is an important methyl mark on proteins is under the direct control of SAM (Trivedi and Deth, 2012) and indirect control of GSH levels. Previous studies have also shown that drugs of abuse can induce histone protein methylation changes, including changes in the heterochromatin transcription levels; e.g. histone lysine methylation marks on LINE-1 are altered after cocaine administration (Maze et al., 2011).

The highly critical yet unanswered puzzle in understanding epigenetic regulation are the factors which determine the probability of CpG-methylation vs. non-CpG-methylation on a DNA strand under a specific cellular environment/a set of various stimuli, including exposure to drugs of abuse (Arand et al., 2012; Guo et al., 2014; Medvedeva et al., 2014). An additional layer of complexity is added by the fact that, a classification pertaining to contributions and sensitivity of the specific gene regions via epigenetic modifications is not yet characterized. Although, in general it is believed that the promoter region of a transcript is highly sensitive and responsive to epigenetic changes, recent evidence indicates that intragenic epigenetic regulation might also be critical for modulating transcriptional status of a gene (Luco et al., 2011). Almost (~50%) of CpG islands (CGIs) are generally present at the promoter regions of annotated genes. However, the other half of CGIs are referred to as “orphan” CGIs, which reflects the unpredictability whether they are present within or between characterized transcription units (Illingworth et al., 2010). Interestingly, the available evidence suggests that many orphan CGIs are also the sites of transcriptional initiation (e.g. CGIs at the 39 end of the *Pomc* gene and exon 2 of the MHC class II I-Ab gene both initiate transcripts of unknown function whose coding potential is minimal Gardiner-Garden and Frommer, 1994; Macleod et al., 1998). Several similar genome-wide analyses have confirmed that a high percentage of orphan CGIs represent novel promoters (Illingworth et al., 2010). However, it is possible that some orphan CGIs probably represent alternative promoters of nearby annotated genes. The latter is highly critical and indicates a novel role of redox and methylation status in stimuli-dependent alternative splicing of a gene variant. Our lab has previously characterized the effects of redox status on alternative splicing of the MS enzyme (Muratore et al., 2013), which provided methionine for use in SAM synthesis. Additionally, we also reported that MS is alternatively spliced in an age-dependent manner as well as in neurodevelopmental and neurodegenerative disorders (Muratore et al., 2013; Hodgson et al., 2013). A number of epigenetic features, including DNA methylation, nucleosome occupancy, specific histone modifications, and protein features, are also strongly associated with alternative splicing (Luco et al., 2011). Thus, CpG sites can affect alternative splicing of genes and are under the direct control of redox status. Although relevant experimental studies are not performed yet, it is plausible that drugs of abuse modulating the redox levels might also influence MS activity. For instance, we have previously reported that opioids alter mRNA levels of the MS enzyme via regulation of GSH-based redox status (Trivedi et al., 2014b).

## Genetic mutations in redox pathways

Although this paper focuses on the redox and epigenetic changes, the role of genetic mutations including single nucleotide polymorphism (SNPs) in a predisposition to abuse illicit drugs should be acknowledged. SNPs in gene sequences responsible for methylation machinery and redox regulators are critical for regulating epigenetic status. Emerging evidence indicates that mutation in DNMT gene sequence is highly critical for the epigenetic homeostasis since these enzymes are directly involved in regulating DNA methylation levels, including *de novo* methylation as well as maintenance methylation. These changes are important during fetal stages (Potter et al., 2013). Even changes in enzymes maintaining the SAM levels can also contribute to the development of a drug addiction phenotype. Methylenetetrahydrofolate reductase (MTHFR) is involved in maintaining methionine synthase activity and indirectly regulates SAM levels. SNP of MTHFR (c.677C > T) can contribute toward the development of alcohol dependence and alcohol's behavioral effects (Saffroy et al., 2008; Shin et al., 2010; Kobayashi et al., 2012). CBS enzyme, which mediates the conversion of homocysteine to cystathionine, can contribute to regulation of epigenetic changes and specifically, SNPs in CBS and MTRR are reported to induce hypermethylation of promoter region in lung epithelial cells of individual's with nicotine addiction (Flores et al., 2012). Morphine and opioid derivatives can induce hypermethylation of promoter region by mediating effects on redox status of neuronal cells (Trivedi et al., 2014a,b). Altered promoter methylation can result either in adaptive transcriptional levels of mRNA or can lead to priming of the gene as discussed in a later section. This can further induce subsequent drug exposure or lead to the development of drug addiction or tolerance. While little work has been performed to qualify this argument, the authors propose that, since the redox/methylation pathway is a key mediator of these global changes in genomic transcriptional and translational status, the first immediate response of cells under any stimuli would revolve around maintaining redox/methylation homeostasis. Hence, the authors reiterate the previously proposed “holonarchy” of several dynamic and intertwined processes starting at the bottom and progressing upward, beginning at protein turnover, post translational modifications, post-transcriptional regulations, transcription modifications, epigenetic changes and the highest level would be GSH-based redox status as a central regulatory switch, not only regulating CpG-methylation but also non-CpG-methylation on mRNA and proteins.

## Gene priming

Chronic drug exposure alters brain gene expression, further inducing long-term structural and functional changes in neural networks, which result in behavioral symptoms that underlie compulsive drug taking and seeking (Sanchis-Segura et al., 2009; Sun et al., 2012). However, the specific mechanisms mediating the translation of drug-induced gene-expression changes and synaptic plasticity into persistent neuroadaptations remains uncharacterized. Intriguingly, preliminary evidence from current investigations on chromatin modulation in drug addiction models suggests that epigenetic modifications at individual genes may not only induce stable changes in mRNA expression of specific genes, but can also alter the “inducibility threshold” of additional genes in response to the same, or some other subsequent stimulus (Robison and Nestler, 2011) (**Figure 4**). Thus, exposure to drugs of abuse or other such stimuli primes the genes in a state which is readily inducible/silenced by subsequent stimuli, although the steady state levels or baseline expression levels of these genes are not affected at that point. While these studies are still in the relatively early phase, the latent epigenetic changes can be viewed as “molecular insults,” which can drastically impinge upon an individual's adaptability, and hence contribute to the behavioral outcomes especially during tolerance and withdrawal phases. More importantly, if these long-lasting changes in chromatin structure are involved in the maintenance of drug addiction behavior, the ability to reverse the epigenetic signature of an addicted state would offer a fundamentally new approach for more effective treatment of drug relapse (Maze and Nestler, 2011).

### Gene priming and drug addiction

Priming effects of cocaine and measuring the epigenetic changes during withdrawal phase or during the subsequent cocaine exposure stages (Levine et al., 2011; Manzanedo et al., 2012; Li et al., 2014) are recently elucidated. In particular, one study identified numerous desensitized genes including transcription of ~10% of genes induced in the NA by acute cocaine exposure, but not induced by a subsequent cocaine challenge after a prior period of chronic exposure to the drug (Maze et al., 2010). On the contrary, some genes were also primed or had poised status, i.e., transcription of genes was not affected by acute cocaine exposure but these genes were induced after a chronic course of cocaine, as evident from approximately three times more genes reported to be elevated in cocaine-experienced animals. Some of these genes had decreased G9a and H3K9me2 at the promoter regions, which suggests a role of epigenetic marks, especially those provided by G9a, in the gene priming effects toward subsequent drug exposure. A minor variant of the above gene priming involves exposure to different drugs, i.e., exposure to a specific drug of abuse followed by a subsequent exposure to a different drug of abuse, and the resulting changes in gene priming, gene expression and epigenetic changes are characterized. One such study by Levine et al. (2011) reported that pretreatment of mice with nicotine increased the response to cocaine, as assessed by addiction-related behaviors, synaptic plasticity adaptations, transcriptional and epigenetic changes in the striatum—a brain region critical for addiction-related behavioral outcomes. It was evident that the cocaine responses were primed by nicotine, since cocaine's ability to induce transcriptional activation of genes (e.g. *FosB*) via histone deacetylase (HDAC) inhibition was enhanced by nicotine, which induced global histone acetylation in the striatum. Previous research has indicated that chronic ethanol feeding and high blood alcohol levels lead to elevated H3K9 acetylation, HAT activity, and p300 HAT (Bardag-Gorce et al., 2007). Noteworthy is the fact that the increased H3K9 acetylation, decreased H3K9 trimethylation and DNA methylation showed strong correlations to changes in gene expression and myeloid body (MB) formation in drug-primed mice. After withdrawal of the drug, gene expression changes and MB formation, as well as the ability of SAM to block this phenomenon, can last up to 4 months (Bardag-Gorce et al., 2007). This most likely represents an epigenetic memory phenomenon of gene expression changes under the effect of drugs of abuse, in this case nicotine, which can further influence the effect of subsequent drug exposure.

### Gene priming and redox status

**Figure 3:**
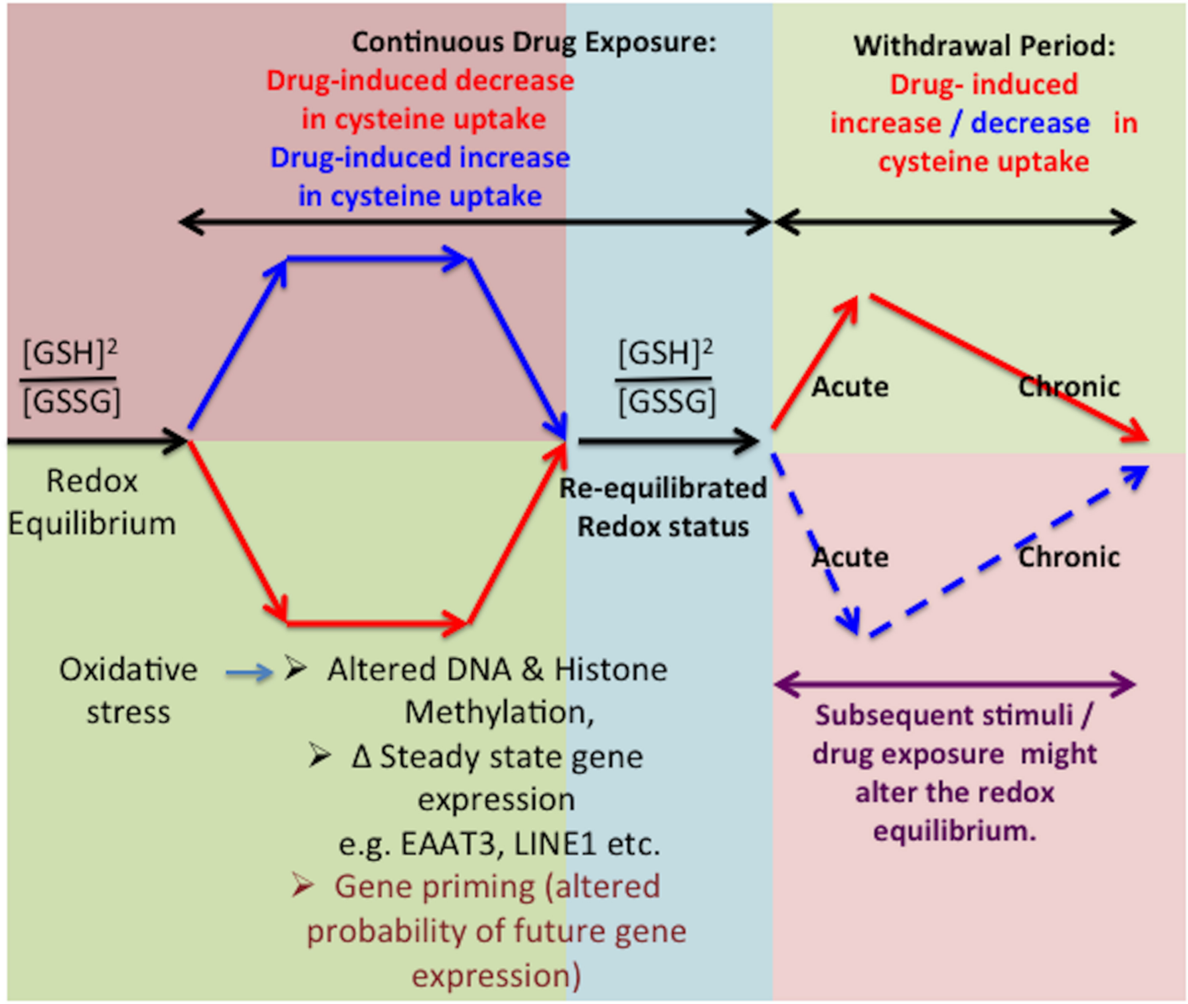
**Effect of *in vitro* washout on redox equilibrium**. Inhibition of cysteine uptake can alter cellular redox potential, resulting in adaptive changes in gene expression via epigenetic effects, which restore the redox equilibrium. However, in the absence of opioids during the washout phenomenon, these adaptive changes can lead to increased cysteine uptake as well as intracellular redox potential. A subsequent drug exposure with same or different drugs of abuse might also induce changes in the redox equilibrium further affecting the transcriptional regulatory mechanisms.

Changes in glutamate transporter expression, mainly GLT1 but also EAAT3, are well-characterized during morphine withdrawal (Xu et al., 2003) and reports indicate no changes in EAAT3-mediated glutamate transport during the withdrawal period. However, these studies did not measure the effects on EAAT3-mediated cysteine transport, and further investigations are needed to define the redox-mediated effects of EAAT3 during the withdrawal period (Xu et al., 2003). The latter could be a period of recovery and compensatory adaptive mechanism during which cells undertake recalibration from the previous insults or stimuli including chronic drug exposure (Christie, 2008). Further, the ability to maintain or restore redox homeostasis might include underlying changes in gene expression mediated via epigenetic-modulation of gene transcription in response to various stimuli, including drugs of abuse (Mao et al., 2002; Xu et al., 2003; McClung et al., 2005; Christie, 2008; Robison and Nestler, 2011; Schwarz et al., 2011). However, we propose that an absence of these stimuli can render the adaptive changes excessive, leading to a plethora of compensatory mechanisms which could potentially result in a switched homeostasis in the opposite direction (Xu et al., 2003, 2006; McClung et al., 2005). Thus, redox-responsive adaptive regulation of epigenetic status might exhibit opposite changes during sustained drug exposure vs. drug withdrawal states and contribute to the observed symptoms (Figure 3).

**Figure 4:**
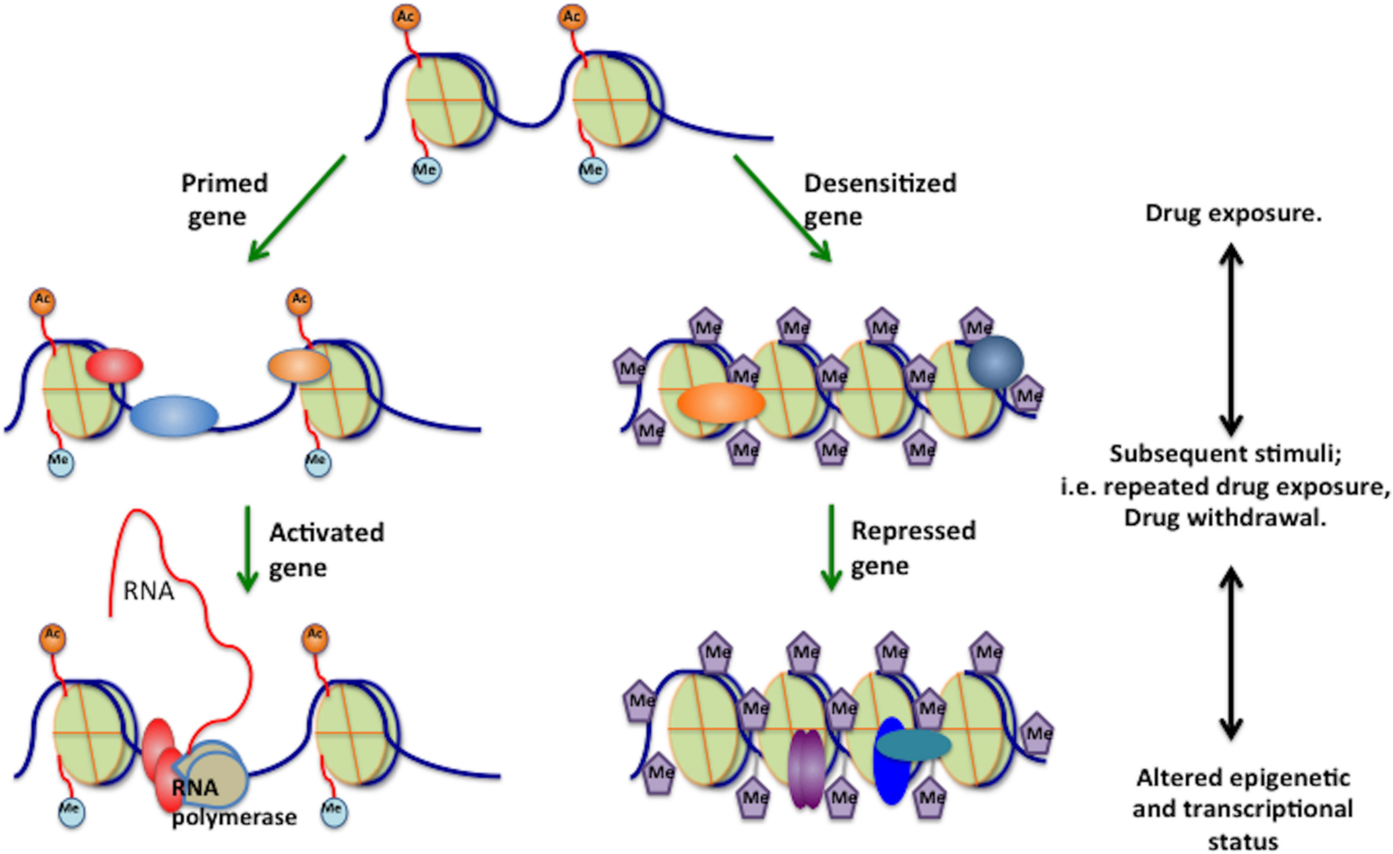
**Gene priming**. Epigenetic mechanisms mediate gene priming and desensitization under the influence of drugs of abuse, and that many of these changes are latent, meaning that they are not reflected by stable changes in steady-state mRNA levels. Instead, these changes would induce subsequent changes in chromatin structure, such that a later drug administration/washout would induce a given gene to a greater (primed) or lesser (desensitized) extent based on the epigenetic modifications induced by previous chronic drug exposure. A, acetylation; M, methylation; P, phosphorylation; pol II, RNA polymerase II.

### Gene priming and drug withdrawal

While gene transcription changes during the withdrawal period have been studied (Spijker et al., 2004; Xu et al., 2006; Christie, 2008), epigenetic mechanisms underlying these changes have not been thoroughly investigated and involvement of changes in redox status in epigenetic and gene transcription changes needs to be explored further. Preliminary *in vivo* results indicate that EAAT3 and GSH levels are affected during withdrawal period (Mazei-Robison and Nestler, 2012). Figure 3 depicts a proposed mechanistic scheme, illustrating contrasting changes in EAAT3-mediated cysteine uptake and redox status during initial drug exposure vs. the withdrawal-related washout period, which can lead to reciprocal changes in gene expression. The key aspect is that adaptive mechanisms operate to maintain redox equilibrium in a homeostatic range. Thus, the redox sensitivity of methylation allows epigenetic regulation of gene transcription to be responsive to the presence of drugs that alter redox status. Since epigenetic effects can be very long-lasting, such changes in transcription serve to extend the consequences of acute drug use and drug withdrawal far beyond the actual period of drug exposure, representing an epigenetic memory of drug abuse. Indeed, some genes involved in compensatory adaptive changes might display gene priming, reflecting graded levels of epigenetic regulation, and transcription-based changes in protein abundance might modify the redox-regulated pathways indicated in Figure 2. Recent advancements (Anker and Carroll, 2011; Robison and Nestler, 2011; Schwarz et al., 2011; Feng and Nestler, 2013; Giannotti et al., 2014; Li et al., 2014) clearly indicate the potential value of investigating the various mechanisms that modulate the reversible transitions between chromatin states under the influence of drug exposure, including gene priming and desensitization.

## Redox replenishers

Recognition of the effects of drugs of abuse on redox and methylation capacity of neuronal cells opens a new therapeutic avenue, which can potentially be exploited to treat or prevent the symptoms associated with drug tolerance and withdrawal; indeed, this is already taking place (Ronis et al., 2005; LaRowe et al., 2006; Chandramani Shivalingappa et al., 2012). Not only are the enzymes and transporters involved in these pathways potential drug targets, but small molecules and metabolites which augment antioxidant status and methylation capacity could regulate behavioral endpoints under the influence of chronic drug exposure (Tian et al., 2012; Anier et al., 2013; McClure et al., 2014). Such agents could act to restore epigenetic status to normal and potentially inhibit the effects of drugs of abuse. In consideration of the above hypothesis, redox-based interventions which replenish antioxidant homeostasis might be useful for abrogating the effects of drugs of abuse on redox-state and methylation capacity, and could be a potential therapeutic strategy for disorders related to illicit drug use.

NAC is the acetylated precursor of L-cysteine, and it protects the amino acid from oxidation in the alimentary tract. NAC is deacetylated after absorption, increasing the levels of intracellular L-cysteine for the synthesis of GSH. NAC can cross the blood brain barrier and studies show that neuronal cell cultures synthesize GSH in proportion to supplied NAC concentrations (Dodd et al., 2008). Due to NAC's neuroprotective action in brain, it has considerable therapeutic potential in treatment of psychiatric disorders like schizophrenia (Lavoie et al., 2008) and bipolar disorder (Dean et al., 2011). NAC is also used clinically for symptomatic treatment of alcoholic patients, cocaine, and heroin abusers (Baker et al., 2003; Mardikian et al., 2007; Zhou and Kalivas, 2008; Ferreira Seiva et al., 2009; Chandramani Shivalingappa et al., 2012). Rodent studies show that ethanol-induced oxidative stress and increased lipid peroxidation can be reversed by NAC intake (Ronis et al., 2005). Several studies measuring oxidative stress status also confirm tha, NAC co-administered with cocaine can prevent a cue-induced desire and relapse to drug craving (Zhou and Kalivas, 2008; Reichel et al., 2011). Several mechanisms have been proposed to account for the actions of NAC in preclinical models of addiction, but this remains unclear. After 1 week of cocaine exposure followed by three subsequent weeks of withdrawal, an IP injection of NAC can recover the decreased basal extracellular glutamate levels in the NA of the cocaine-treated rats (Baker et al., 2003). It is noteworthy that when the Xc^−^ transporter is inhibited, the NAC-induced recovery of extracellular glutamate levels in NA is also inhibited, indicating that the Xc^−^ system may be important in the neurobiological mechanisms of drug addiction (Kim et al., 2003). Moreover, these results suggest that NAC can potentially induce recovery of down-regulated Xc^−^ and GLT-1 function (Dodd et al., 2008; Knackstedt et al., 2009; Reissner et al., 2014). This would normalize glutamate homeostasis, which is important for long term potentiation and memory formation and is implicated in drug addiction, especially drug tolerance and withdrawal (Knackstedt et al., 2009, 2010; Knackstedt and Kalivas, 2009). Indeed, knockdown of GLT-1 in the NA interferes with the ability of NAC to inhibit reinstatement of cocaine self-administration and this effect was blocked by a mGluR5 antagonist, suggesting that increased glutamate plays an essential role (Reissner et al., 2014; Scofield and Kalivas, 2014). In addition to protecting brain cells against oxidative stress, GSH also enhances NMDA receptor response to glutamatergic stimulation, indicating that glutamatergic signaling could potentially be regulated in response to NAC administration (Dean et al., 2011; Penugonda and Ercal, 2011; Schmaal et al., 2012; McClure et al., 2014). NAC is reported to prevent relapse behaviors, reducing drug-associated cues-, cocaine-, and heroin-priming-induced reinstatement after extinction as well as following abstinence protocols (Baker et al., 2003; LaRowe et al., 2006; Dean et al., 2011; Reichel et al., 2011). In addition, even the hangover effects produced by a large ingestion of alcohol can be reversed by NAC (Ronis et al., 2005), the mechanism for which is still unknown.

A different approach is provided by ceftriaxone, a beta-lactam antibiotic, which up-regulates both GLT-1 and xC^−^ (Alhaddad et al., 2014b; Rao and Sari, 2014), which is associated with decreased ethanol consumption by rats with an alcohol preference (Sari et al., 2013; Alhaddad et al., 2014a). MS-153, a synthetic agent, that increases GLT-1-mediated glutamate transport (Shimada et al., 1999), can decrease alcohol (Alhaddad et al., 2014b), morphine, cocaine, and amphetamine consumption (Nakagawa et al., 2001, 2005). A recent study found that MS-153 up-regulates GLT-1 levels in NA, but not in the prefrontal cortex (Alhaddad et al., 2014b). Taken together, these studies indicate that modulation of glutamate and/or cysteine/cystine status holds potential for treatment of drug abuse.

Cobalamin is the cofactor of methionine synthase, which accepts a methyl-group from 5-methyltetrahydrofolate (5-MTHF) and adds it to homocysteine to form methionine. During oxidative stress Cbl(I), the oxidation state of cobalamin that readily forms methylcobalamin (MeCbl), will oxidize to Cbl(II), which cannot be methylated leading to oxidative stress which inhibits MS activity. Such oxidation of cobalamin represents a redox-sensitive switch for controlling the methylation cycle, and MeCbl regulates MS activity (Deth et al., 2008). Provision of exogenous MeCbl can increase MS activity and sustain a higher level of methylation. During oxidative stress, MS is inhibited to prevent HCY conversion to methionine, shifting HCY to the transulfuration pathway for synthesis of GSH so as to maintain cellular antioxidant defenses against oxidative stress (Lu et al., 2000; Muratore et al., 2013). Restoration of adequate GSH levels promotes formation of GSCbl and MeCbl, leading to MS reactivation. Hence, drugs of abuse decrease GSH level, compensatory mechanisms can potentially lead to decreased synthesis of methionine from HCY and an increase of HCY flux toward transulfuration and the maintenance of GSH levels. This decrease in MS activity induces a decrease in SAM and reduces methylation capacity, causing global DNA (Benzecry et al., 2004; Muratore et al., 2013) and histone hypomethylation. The addition of MeCbl could potentially prevent these effects by promoting MS activity, abrogating the usual redox-dependent epigenetic changes and potentially preventing the epigenetic memory induced under the effects of drug exposure as well as drug-mediated transcriptional and behavioral changes. To our knowledge, no studies have been conducted to identify or characterize the effects of MeCbl on drug addiction, or withdrawal phenomenon, and hence, it is a novel therapeutic avenue remaining to be explored.

## Conclusion

**Figure 5:**
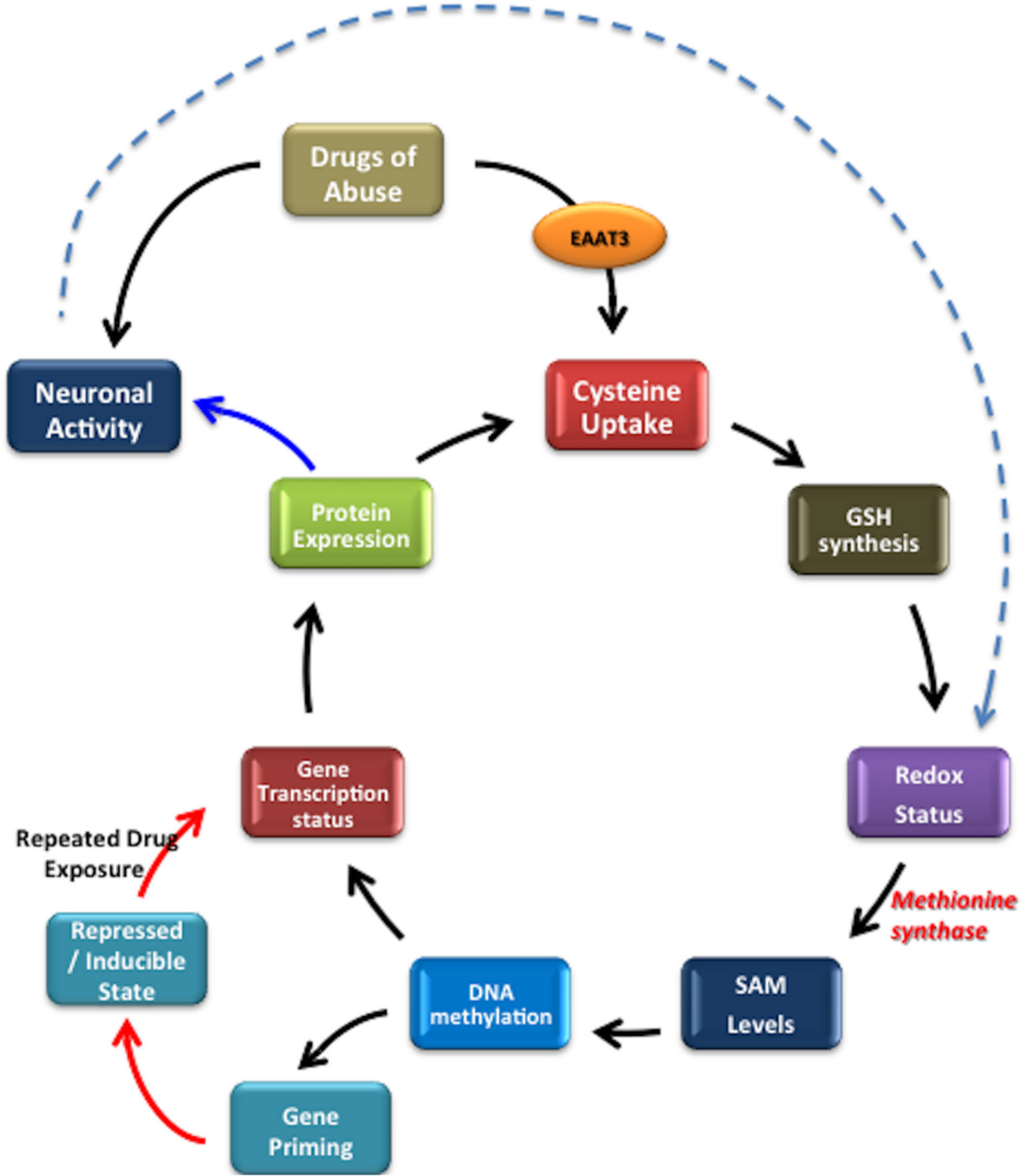
**Summary: Redox-based epigenetic signaling**. Drugs of abuse alter cysteine uptake, affecting GSH synthesis, which shifts redox status and the reducing potential. This shift affects methionine synthase (MS) activity further affecting the probability of DNA methylation i.e., epigenetic changes, subsequently inducing changes in gene transcription or gene inducibility. If a repeated stimuli occurs, it results in altered gene transcription. This eventually affects the protein expression and consequently neuronal functionality and phenotype. Dotted line indicates that, neuronal activity can also redox status by altering the levels of ROS produced.

Based on the observations from the above studies, we propose a novel redox-based epigenetics mechanism contributing to drug addiction (Figure 5). The resultant stochastic nature of changes in the chromatin structure emphasizes the research needed to identify specific set of genes, which are altered at the steady state mRNA levels as well as those genes which are epigenetically primed, during drug exposure. Altered redox homeostasis contributes as a driving force to mediate these changes. Furthermore, focused research is also needed for redox-based or methylation-based interventions, which can potentially be used to reverse the effects of drugs of abuse and used for drug addiction treatment.

### Conflict of interest statement

The authors declare that the research was conducted in the absence of any commercial or financial relationships that could be construed as a potential conflict of interest.
